# Distribution of glycine receptors on the surface of the mature calyx of Held nerve terminal

**DOI:** 10.3389/fncir.2014.00120

**Published:** 2014-10-06

**Authors:** Johana Trojanova, Akos Kulik, Jiri Janacek, Michaela Kralikova, Josef Syka, Rostislav Turecek

**Affiliations:** ^1^Department of Auditory Neuroscience, Laboratory of Synaptic Transmission, Institute of Experimental Medicine, Academy of Sciences of the Czech RepublicPrague, Czech Republic; ^2^Department of Physiology II, University of FreiburgFreiburg, Germany; ^3^BIOSS Centre for Biological Signalling Studies, University of FreiburgFreiburg, Germany; ^4^Department of Biomathematics, Institute of Physiology, Academy of Sciences of the Czech RepublicPrague, Czech Republic

**Keywords:** presynaptic, glycine receptor, MNTB, calyx of Held, pre-embedding immunoelectron microscopy, spillover

## Abstract

The physiological functions of glycine receptors (GlyRs) depend on their subcellular locations. In axonal terminals of the central neurons, GlyRs trigger a slow facilitation of presynaptic transmitter release; however, their spatial relationship to the release sites is not known. In this study, we examined the distribution of GlyRs in the rat glutamatergic calyx of Held nerve terminal using high-resolution pre-embedding immunoelectron microscopy. We performed a quantitative analysis of GlyR-associated immunogold (IG) labeling in 3D reconstructed calyceal segments. A variable density of IG particles and their putative accumulations, inferred from the frequency distribution of inter-IG distances, indicated a non-uniform distribution of the receptors in the calyx. Subsequently, increased densities of IG particles were found in calyceal swellings, structures characterized by extensive exocytosis of glutamate. In swellings as well as in larger calyceal stalks, IG particles did not tend to accumulate near the glutamate releasing zones. On the other hand, GlyRs in swellings (but not in stalks) preferentially occupied membrane regions, unconnected to postsynaptic cells and presumably accessible by ambient glycine. Furthermore, the sites with increased GlyR concentrations were found in swellings tightly juxtaposed with GABA/glycinergic nerve endings. Thus, the results support the concept of an indirect mechanism underlying the modulatory effects of calyceal GlyRs, activated by glycine spillover. We also suggest the existence of an activity-dependent mechanism regulating the surface distribution of α homomeric GlyRs in axonal terminals of central neurons.

## Introduction

The subcellular distribution of ligand-gated ion channels (LGICs) in neuronal cells is tightly correlated with the physiological functions of the receptors. Somatodendritic receptors reside at subsynaptic sites, generating fast and phasic responses to synaptic transmitters, as well as in extrasynaptic compartments, where they mediate slow or tonic modulation of neuronal activity (Farrant and Nusser, 2005; Muller et al., 2008; Hardingham and Bading, 2010; Vizi et al., 2010; Brickley and Mody, 2012; Kopach and Voitenko, 2013). Numerous immunohistochemistry examinations have shown that synaptic receptors typically form intrasynaptic clusters while extrasynaptic receptors are mostly dispersed (Bernard et al., 1997; Caruncho et al., 1997; Kharazia and Weinberg, 1997; Nusser et al., 1998; Walmsley et al., 1998; Zarei et al., 1999; Kieval et al., 2001; Rubio and Soto, 2001; Geiman et al., 2002; Wei et al., 2003; Masugi-Tokita et al., 2007; Petralia, 2012). Little is known about the localization of LGIC in the plasma membrane of presynaptic nerve terminals. These receptors mediate relatively slow modulation of presynaptic exocytosis and plasticity, and their physiological activation often results from the spillover of neurotransmitters (Danbolt, 2001; Kullmann, 2001; Boehm and Kubista, 2002; Engelman and MacDermott, 2004; Pinheiro and Mulle, 2008; Trigo et al., 2008; Verhoog and Mansvelder, 2011). The surface distribution of LGIC in presynaptic nerve terminals would therefore be expected to be similar to that of extrasynaptic receptors in somatodendritic compartments (Kieval et al., 2001; Belenky et al., 2003; Darstein et al., 2003; Ruiz et al., 2003; Jourdain et al., 2007).

Receptors for inhibitory neurotransmitter glycine form chloride permeable ion channels and belong to the Cys-loop family of LGIC (Lester et al., 2004). Native GlyRs are expressed in both pre- and postsynaptic parts of mature neurons. GlyRs on nerve terminals form homomers of α1 subunits which disperse onto the presynaptic plasma membrane (Turecek and Trussell, 2002; Jeong et al., 2003; Deleuze et al., 2005; Morkve and Hartveit, 2009; Kubota et al., 2010; Hruskova et al., 2012; Xiong et al., 2014) and mediate a slow modulation of neurotransmitter release (Turecek and Trussell, 2001; Jeong et al., 2003; Chu et al., 2012; Hruskova et al., 2012). GlyRs potentiate exocytosis of glutamate in the calyx of Held, a large axonal terminal of globular bushy cells located in the anteroventral cochlear nucleus and projecting to principal cells (PC) in the medial nucleus of trapezoid body (MNTB). Physiological activation of calyceal GlyRs involves heterosynaptic cross-talk and/or glycine spillover from surrounding glial cells (Turecek and Trussell, 2001; Kopp-Scheinpflug et al., 2008). A mature calyx of Held is comprised of morphologically and functionally distinct parts (Rowland et al., 2000; Wimmer et al., 2006) suggesting an appropriate compartmentalization of calyceal GlyRs. However, the subcellular distribution of GlyRs in the calyx has not yet been reported. By using high resolution immunoelectron microscopy, we show that GlyRs occur in calyceal swellings and stalks, compartments responsible for glutamate release. Moreover, the data show that GlyRs are not just randomly dispersed on the surface of calyx and that their localization pattern depends on the presence of endogenous sources of agonists.

## Materials and methods

### Animals

Experiments were performed on three adult male Wistar rats (~250 g) obtained from Charles River, Freiburg, Germany or Institute of Physiology, ASCR, Prague, Czech Republic. The care and handling of animals before and during the experimental procedures followed European Union regulations and was approved by the Animal Care and Use Committees of the authors’ institutions.

### Tissue preparation

Animals were deeply anesthetized using ketamine-xylazin (100 mg/kg, 16 mg/kg body weight; Calypsol, Gedeon Richter, Hungary; Xylapan, Vétoquiol, UK) and perfused transcardially with 0.9% saline followed by a fixative containing 4% (w/v) paraformaldehyde (Sigma-Aldrich, USA), 15% (v/v) picric acid (saturated aqueous solution; 1.3% in H_2_O; Sigma-Aldrich, USA) and 0.05% (v/v) glutaraldehyde (conc. 25%; TAAB, UK) in 0.1 M phosphate buffer (PB). Brains were excised, postfixed, washed in PB, and 50 µm thick coronal sections were cut using the VT100S slicer (Leica, Germany).

### Pre-embedding immunoelectron microscopy

Brainstem sections were cryoprotected in solution containing 25% (w/v) sucrose and 10% (v/v) glycerol in 50 mM PB. The sections were freeze-thawed and incubated in a blocking solution containing 20% (v/v) Chemiblocker (Chemicon, Millipore, USA) in 50 mM Tris-buffered saline (TBS, pH 7.4) for 4 h, followed by incubation with primary antibodies diluted in TBS containing 5% (v/v) Chemiblocker overnight at 4°C. We used polyclonal rabbit antibodies recognizing the second intracellular loop of the α1 subunit of GlyR (0.75 µg/ml) or vesicular GABA transporter (vGAT; 1.7 µg/ml), and polyclonal guinea pig antibodies, against vesicular glutamate transporter 1 (vGluT1; 1.25 µg/ml) (all from Synaptic Systems, Germany). The sections were then incubated in a mixture of biotinylated goat anti-guinea pig IgG antibody (Jackson ImmunoResearch Laboratories, USA) and goat anti-rabbit IgG antibodies coupled to 1.4 nm gold particles (Nanoprobes, USA) overnight at 4°C. After several washes in 25 mM phosphate-buffered saline (PBS), the sections were post-fixed in 1% (v/v) glutaraldehyde in PBS, followed by intensification with HQ Silver Enhancement kit (Nanoprobes) and then incubation in the Vectastain ABC Kit (Vector Laboratories, USA). Sections were further treated with 1% osmium tetroxide (TAAB, UK), stained with 1% (w/v) uranyl acetate (Polysciences, USA), dehydrated in a graded series of ethanol and propylene oxid (Polysciences), and flat-embedded in epoxy resin (Durcupan ACM, Sigma-Aldrich, Gillingham, UK). After polymerization, 70–80 nm thick sections were cut using an ultramicrotome Reichert Ultracut S (Leica, Germany). The slices were examined using the LEO 906E transmission electron microscope (Carl Zeiss, Germany). Images were acquired by BioVision/VarioVision 3.2 software (Soft Imaging System; Olympus).

### Analysis of GlyR immunolabeling

**Figure 1:**
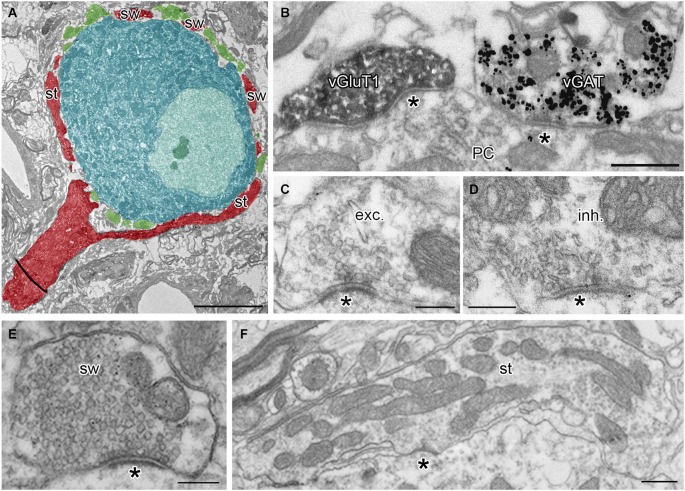
**Identification of presynaptic terminals and postsynaptic neurons in adult MNTB**. **(A)** A single ultrathin section through the calyx of Held terminal (red) and inhibitory boutons (green) surrounding the soma of the MNTB PC (blue). Note that the pre-calyceal axon branches to large stalks (st) and smaller calyceal swellings (sw). **(B)** An electron micrograph showing a calyceal process, immunostained for vGluT1 (peroxidase reaction end product), and an inhibitory bouton, immunopositive for vGAT (gold particles). Asterisks indicate synaptic contacts between the calyx or the inhibitory ending and a PC. **(C,D)** Examples of putative excitatory (exc.) and putative inhibitory (inh.) active zones (AZ) at synaptic contacts (asterisk) formed by the calyceal or presumed inhibitory nerve terminals on somata of the PC. Note round vs. pleomorphic synaptic vesicles in excitatory vs. inhibitory terminals. **(E,F)** Electron micrographs of cross-sections through calyceal processes considered as swelling (sw) or stalk (st). Scale bars: 5 µm **(A)**, 0.5 µm **(B,F)**, 0.2 µm **(C,D,E)**.

**Figure 2:**
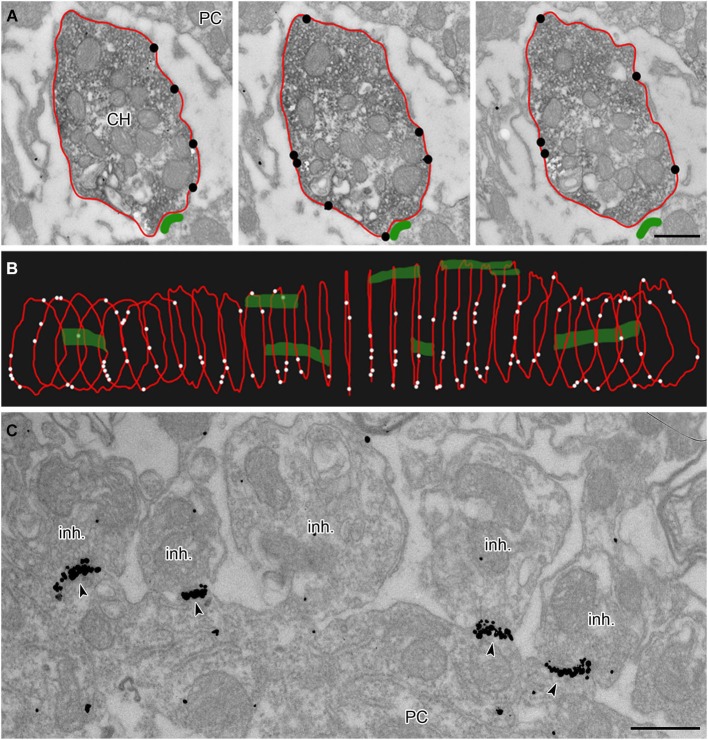
**Analysis of membrane-localized GlyR alpha1-immunoreactivity in 3D reconstructed calyceal processes**. **(A)** Three consecutive ultrathin sections through a calyceal swelling (CH) and a PC. The calyx is immunostained for vGluT1 (peroxidase) and for GlyR α1 (IG particles; indicated by black circles) along the presynaptic plasma membrane (outlined red). The postsynaptic density (PSD) of synaptic contacts between the calyx and PC are labeled green. **(B)** 3D alignment of digital contours of 29 serial sections (each 70 nm thick) of the same calyceal segment as in **(A)**, showing the dispersed distribution of presynaptic GlyRs (white dots) and synaptic contacts (green). **(C)** Electron micrograph showing clusters of GlyR-associated IG particles (arrowheads) at postsynaptic sites juxtaposed with putative inhibitory terminals (inh.). Scale bars: 0.5 µm **(A,C)**.

Calyces of Held were identified as vGluT1 positive structures containing round synaptic vesicles and forming asymmetric synaptic contacts with MNTB PC (Figures 1A,B,C; Billups, 2005). Calyceal stalks and swellings were distinguished based on their different morphological properties. Stalks were identified as first order branches of the myelin-free pre-terminal axons (also called axonal heminodes; Leão et al., 2005), extending over the surface of postsynaptic neuron (Rowland et al., 2000; Sätzler et al., 2002; Figures 1A,F). Swellings were smaller processes with round cross sections containing numerous synaptic vesicles (Rowland et al., 2000; Wimmer et al., 2006; Figures 1A,E). Inhibitory nerve terminals were distinguished as vGluT1-immunonegative and vGAT-immunoreactive boutons that contained pleomorphic vesicles and formed symmetric synaptic contacts with a PC (Figures 1B,D; Hruskova et al., 2012). Plasma membrane GlyR-associated IG labeling was analyzed in electron micrographs using ImageJ software,^1^ Reconstruct™  software (Fiala, 2005) and IRIS Explorer (NAG, UK). We analyzed images of 2304 cross-sections through calyceal processes. The presynaptic plasma membrane surrounding each section was depicted with a contour line (Figure 2A; Tamaru et al., 2001). 11–69 contoured serial sections were used to compile each of the 72 calyceal segments in total (an example of a segment is shown in Figure 2B). The surface area of a segment was estimated using the Cavalieri principle (Duerstock et al., 2003). All data are presented as mean ± SD. Statistical significance was assessed using the non-parametric two-tailed Mann-Whitney test, two-tailed Kolmogorov-Smirnov test, unpaired and paired two-tailed Students’s *t*-tests or one-way ANOVA followed by Dunnett’s or Bonferroni’s multiple comparison test, using Prism 5.04 (GraphPad, USA) or XLStat 2014.4.07 (Addinsoft).

## Results

### Non-uniform distribution of GlyRs at the calyx of Held nerve terminal

**Figure 3:**
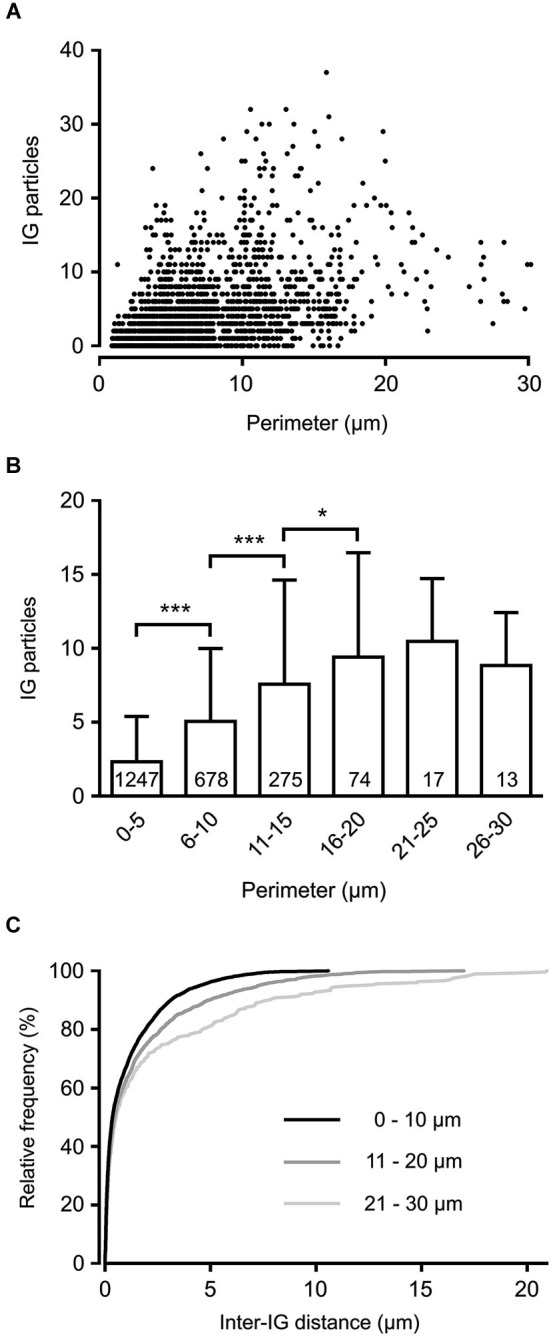
**Non-homogenous distribution of GlyRs in the calyx of Held terminal**. **(A)** The plot shows IG particle numbers found in variously sized calyceal cross-sections (*N* = 2304). Each perimeter value is derived from the length of a contour line drawn along the presynaptic plasma membrane (as illustrated in Figure 2A). **(B)** Bar graph comparing IG particle numbers in sections sorted into six groups based on their perimeter length. *** *P* < 0.001; * *P* < 0.05; Bonferroni’s multiple comparison test. The number inside each column indicates sample size of the group. **(C)** Cumulative histograms of inter-IG distances measured in the sections sorted into three size groups as indicated. Note that shorter inter-IG distances dominate in all distributions.

To reveal the surface distribution of presynaptic GlyRs, we performed a quantitative analysis of their plasma membrane-associated labeling with anti-GlyRα1-coupled IG particles in calyceal segments (as illustrated in Figures 2A,B). Presynaptic IG particles appeared to have a dispersed distribution (see an example in Figure 2B) which contrasted with a clustered distribution of the particles at postsynaptic sites (Figure 2C). The latter staining pattern is typical for somatodendritic GlyRs that accumulate in synaptic contacts between inhibitory boutons and MNTB PC (Hruskova et al., 2012). The average surface density of the presynaptic IG particles varied from 0.4 to 39.1 per µm^2^ (mean ± S.D. = 9.3 ± 7.2 IG/µm^2^, *N* = 72 segments). The calyx of Held is a complex structure comprising of larger stalks that branch via tiny necks into swellings of various sizes (Rowland et al., 2000; Perkins et al., 2010). To assess the distribution of labeling among differently sized parts of the compartments, we plotted the number of IG particles on each calyceal cross-section against its perimeter length (Figure 3A). The number of the particles roughly correlated with the size of each section. A sorting of sections into the six size groups revealed that the number of IG particles increases until the perimeters reach the range of 16–20 µm and then it remains similar in sections with perimeters >20 µm (Figure 3B). This implies lower average densities of IG particles in larger sections and suggests that calyces do not have a simply random distribution of GlyRs. To further test this, we measured distances between IG particles (inter-IG distances) along the plasma membrane and compared them across the three sectional size groups (Figure 3C). Cumulative frequency histograms of the distances for each group indicate that shorter inter-IG distances (<2 µm) dominate in about 70% of the sections, irrespective of their size. Thus the results indicate sites with increased densities of IG particles implying a preferential localization of GlyRs at some compartments of the calyx.

### Increased density of GlyRs in calyceal swellings

**Figure 4:**
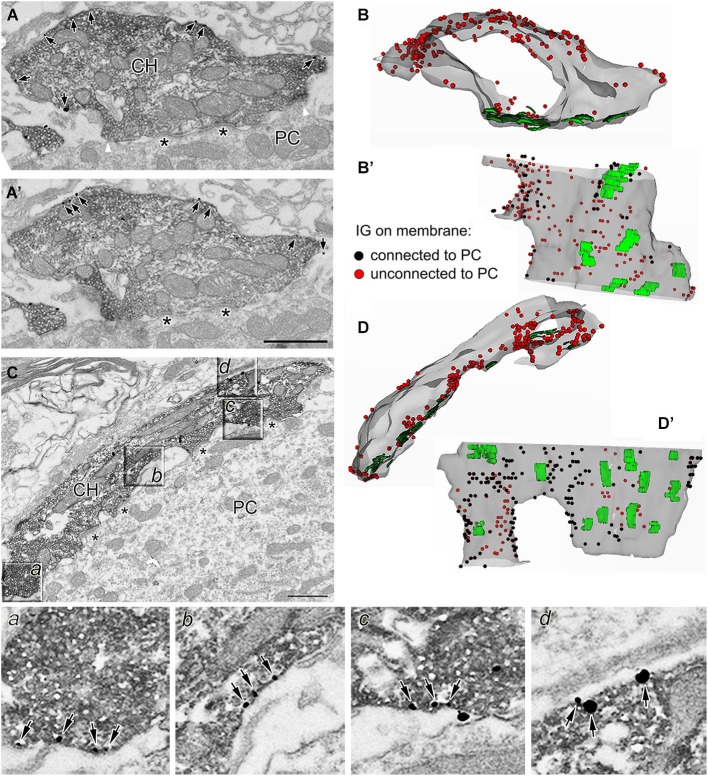
**Localization of GlyRs in identified calyceal processes**. **(A,A’)** Two consecutive sections through a calyceal swelling (CH) double labeled with antibodies against vGluT1 (peroxidase) and GlyR α1 (IG particles; arrows). Asterisks denote synaptic contacts between the calyx and a PC. White arrowheads in panel A indicate borders between membrane regions that are either connected or unconnected to the soma of a PC. **(B)** A semitransparent superposition of 29 contoured sections as in **(A)** to reconstruct a segment of swelling with several synaptic junctions (green areas) and numerous GlyRs (red dots). **(B’)** The same stack of contoured sections as in **(B)** viewed from a different angle (the PC facing side). Black and red dots illustrate GlyRs localized in the calyceal membrane that is either connected or unconnected to the soma of a PC. **(C)** EM image of anti-vGluT1 labeled calyceal stalk immunoreactive for GlyR α1. Sectors in boxes **(a–d)**, displayed on an expanded scale below, show examples of GlyRs (arrows) on plasma membrane that is unconnected **(a,d)** to the postsynaptic cell or that encloses extended extracellular spaces **(b,c)**. **(D)** The 3D reconstruction of 28 serial sections as in **(C)** illustrating a semitransparent segment of calyceal stalk forming synaptic contacts (green areas) with a PC and containing numerous GlyRs (red dots). **(D’)** The same stack of contours as in **(D)** viewed from a different angle (the PC facing side). Scale bar: 1 µm **(A’,C)**.

Morphologically distinct calyceal compartments were found to differ in number and composition of presynaptic ion channels (Dodson et al., 2003; Elezgarai et al., 2003; Leão et al., 2005; Spirou et al., 2008). We next tested whether two main calyceal structures, stalks and swellings, responsible for the release of glutamate, contain different amounts of presynaptic GlyRs. Calyceal segments were reconstituted using 11–69 (33 on average) serial sections through each identified stalk (*N* = 11) (Figures 4C,D,D’) or swelling (*N* = 51) (Figures 4A,A’,B,B’). To estimate the surface density of GlyRs, the number of membrane localized IG particles was counted in each segment and normalized to its surface area value. We found a higher relative density of the particles in swellings (10.8 ± 7.5 IG/µm^2^) compared to stalks (5.9 ± 2.7 IG/µm^2^, *P* < 0.001; unpaired Student’s *t*-test with Welch’s correction for unequal variances) indicating that GlyRs are more frequent in swellings than in stalks.

**Figure 5:**
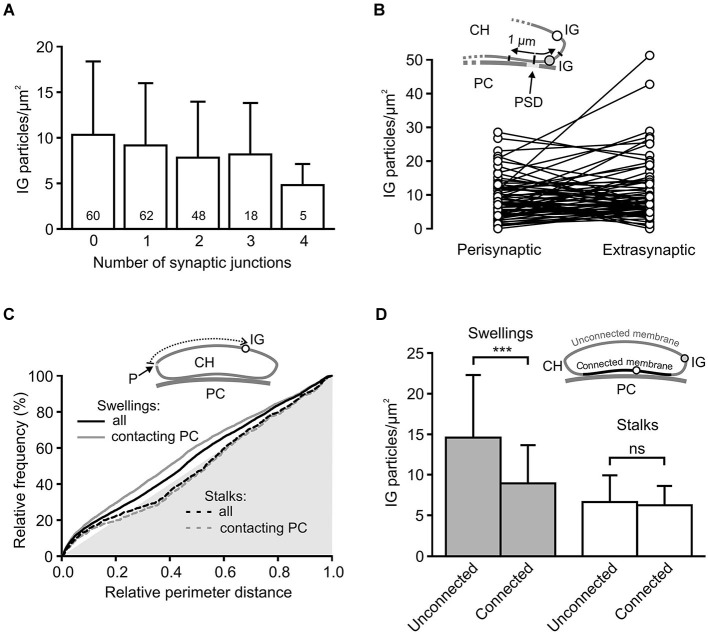
**Differential distribution of GlyRs in calyceal swellings and stalks. (A)** Bar graph showing the average densities of IG particles in calyceal subsegments containing various numbers (0–4) of synaptic contacts with a PC (see Results). The differences between the groups are not significant (data from all 72 calyceal segments; Bonferroni’s Multiple Comparison Test). Numbers inside the columns indicate sample size for each group. **(B)** The plot compares densities of IG particles that lie in two 1 µm-long membrane parts, left and right from the middle of PSD (Perisynaptic), and those that are distributed in remaining parts of sections (Extrasynaptic) constituting subsegments (*N* = 71) containing a single synaptic junction. Sections without any IG particles were excluded from the analysis. Inset illustrates a part of the calyx of Held (CH) process that is apposed to the PSD of the PC and is bearing extrasynaptic (empty circle) and perisynaptic (gray circle) IG particles (IG). **(C)** Cumulative histograms of distances between IG particles and a reference point (P) to compare IG particle distribution along perimeters of sections through stalks (dashed lines) or swellings (solid lines). The reference point was arbitrarily set at the left horizontal edge of a section and the distances were measured in a clockwise direction (see scheme in inset). The histograms were constructed using unbinned data either from all sections (black lines) constituting 51 swellings (6746 distances from 1101 sections) and 11 stalks (1425 distances from 236 sections) or from a selection of sections in which calyceal processes clearly contacted somata of PC (gray lines; swellings: 4572 distances from 628 sections; stalks: 1063 distances from 202 sections) while sections without the contacts were excluded. In all histograms, distributions of IG particles are not random as they significantly differ from the distribution of perimeter distances of surface points, which lie on the hypotenuse of the gray triangle (*P* < 0.001 for all distributions; Kolmogorov-Smirnov test). The exclusion of sections without the contacts led to significant changes in the distribution for swellings (solid gray line; *P* < 0.001; Kolmogorov-Smirnov test) but not for stalks (dashed gray line; Kolmogorov-Smirnov test). **(D)** Bar graph comparing the average densities of IG particles in two regions of the segments from swellings (gray; *N* = 36) and stalks (empty; *N* = 11). The plasma membrane of each section was divided into two parts: membrane connected or unconnected to the soma of a PC (as illustrated by scheme in inset; IG particle in the connected or unconnected membrane is indicated by empty or gray circle). *** *P* < 0.001; paired *t*-test.

Some of the presynaptic LGICs were localized near the neurotransmitter releasing active zones (AZ; Jaskolski et al., 2005; Pinheiro and Mulle, 2008; Trigo et al., 2010). Consistent with this, the 3D reconstructions of calyceal stalks and swellings show numerous IG particles near the synaptic junctions (Figures 4B’,D’). As noted previously (Hermida et al., 2010), calyceal swellings form more synaptic contacts with the postsynaptic cell than stalks do. It is therefore possible that an accumulation of GlyRs over the glutamatergic AZ accounts for the more frequent occurrence of the receptors in the swellings. To test this, we compared densities of IG particles in calyceal segments containing various numbers of synaptic junctions. Consistent with the literature we found more junctions in swellings (0.4 ± 0.1 per µm^2^) than in stalks (0.3 ± 0.2 per µm^2^, *P* < 0.05; unpaired Student’s *t*-test). Serial sections of each calyceal segment were then sorted into five groups based on the number of synaptic junctions (0–4) and IG particles were counted in these subsegments. As shown in Figure 5A, IG particle density was similar between the groups indicating that the number of GlyRs did not correlate with the amount of junctions in the subsegments. Furthermore, we tested whether GlyRs show preferential location around the AZ. Perisynaptic heteroreceptors are observed to lie within tens to hundreds of nanometers from the edges of synaptic contacts (Jones and Wonnacott, 2004; Nyíri et al., 2005; Paspalas and Goldman-Rakic, 2005). The length of the postsynaptic density (PSD) in sections through the calyx of Held synapses appears to be quite variable and spans the range of several hundreds of nanometers (Rowland et al., 2000; Sätzler et al., 2002; Taschenberger et al., 2002; Hermida et al., 2010). To estimate proportions of GlyRs spatially related to the AZ, we therefore compared IG particle densities in 2 µm-long, PSD containing membrane regions (left and right borders of the region were placed 1 µm aside from the middle of PSD; see inset in Figure 5B), and in the rest of the membrane in sections containing a single synaptic contact. The data in Figure 5B show that the relative amounts of particles in membrane parts adjacent to an AZ (Perisynaptic) are not significantly different from the amounts found in more distant parts (Extrasynaptic) (9.0 ± 6.2 IG/µm^2^ vs. 10.8 ± 9.2 IG/µm^2^; paired Student’s *t*-test). Thus the results do not indicate an elevated intrasynaptic or perisynaptic IG labeling, which argues against the possibility that a higher density of GlyRs in swellings is caused by their preferential location around the AZ.

The 3D reconstructions shown in Figure 4 also suggest that IG particles could distribute on the surface of swellings and stalks differently. In swellings, IG particles appear to be more frequent on the side which is not contacting the postsynaptic soma (Figures 4B,B’). In stalks, the particles seem to prefer the side which is facing the PC (Figures 4D,D’). To test this, we compared distributions of IG particles along the perimeters of cross-sections obtained from stalks and swellings. In each section we measured perimeter distances between every IG particle and a point which was arbitrarily set at the horizontal edge of a section (see inset in Figure 5C). The cumulative frequency distributions of the distances normalized against the perimeter length are shown in Figure 5C (black lines). Differences between the histograms are consistent with the assumption that in swellings, IG particles occupy more frequently locations that roughly correspond to the plasma membrane that is not in contact with the postsynaptic cell (*P* < 0.001; Kolmogorov-Smirnov test). This is supported by more pronounced difference between the distributions after exclusion of sections without clear contacts between calyces and PC (Figure 5C, gray lines).

The assumption was further tested by counting IG particles in two parts of the surface of calyceal segments (see inset in Figure 5D). The first part comprised the plasma membranes that contacted the postsynaptic cell body (including those enclosing the extended extracellular spaces; Rowland et al., 2000) while the second part included membranes that were not in contact with the postsynaptic cell. The borders separating these membrane parts are indicated by white arrowheads in Figure 4A. As shown in Figure 5D, IG particle density was significantly higher in the latter part of the surface of swellings but not of stalks. Thus the data support a differential distribution of GlyRs in stalks and swellings and suggest that increased densities of GlyRs in calyceal swellings could at least partially be explained by an accumulation of the receptors at the side that faces away from the body of the MNTB principal neuron.

### Accumulation of GlyRs in swellings in close proximity to glycinergic boutons

**Figure 6:**
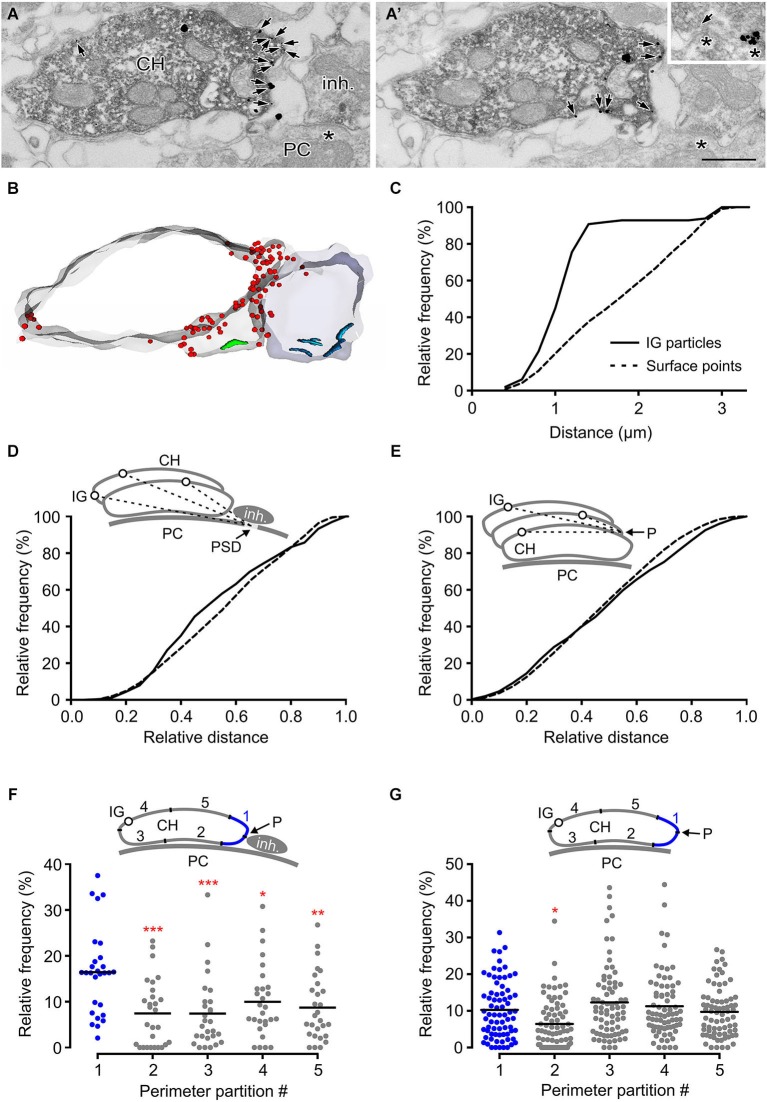
**GlyRs tend to accumulate in calyceal swellings proximal to inhibitory boutons. (A,A’)** Two consecutive sections through a calyceal swelling (CH), immunoreactive for vGluT1 (peroxidase) and GlyR α1 (IG particles; arrows), and vGluT1-immunonegative, putatively inhibitory bouton (inh.). Asterisks denote synaptic contacts between the bouton and a PC. Inset shows an example of a flat vesicle (arrow) at the inhibitory AZ and a cluster of IG particles at the inhibitory synaptic contact. Scale bar: 0.5 µm. **(B)** Stack of contours from 17 serial EM images of sections shown in **(A)**. Note an accumulation of GlyRs (red dots) in calyceal compartments adjacent to the bouton. Synaptic contacts made by the calyx or by the bouton with a PC are shown in green or blue. **(C)** Cumulative histogram showing the distribution of spatial distances between each IG particle (solid line) on the calyceal segment shown in **(B)** and the middle of the PSD of the nearest synaptic contact between the inhibitory bouton and a PC (see inset in panel **D**). For comparison, the distribution of the distances between digitized points delineating the surface of the swelling and the middle of the PSD is shown (dotted line). The distributions significantly differ (*P* < 0.001; Kolmogorov-Smirnov test; bin width = 0.2 µm). **(D)** The distribution of distances provided by similar measurements as in **(C)**. The data was collected from 12 segments of swellings adjacent to an inhibitory bouton. The lengths are plotted relative to the longest distance in a segment. The distributions of IG particles and surface points are significantly different (*P* < 0.001; Kolmogorov-Smirnov test; bin width = 0.05). **(E)** The histogram shows spatial distances between each IG particle (solid line) or each digitized surface point (dotted line) of a calyceal segment and a reference point (P) which was arbitrarily set at the horizontal edge of a section that is placed in the middle of the segment (see inset).The data was collected from 21 swellings without any detectable inhibitory bouton in their proximity. Note that the distribution of IG particles in these segments is not random (*P* < 0.001; Kolmogorov-Smirnov test; bin width = 0.05). **(F,G)** The plots show relative frequencies of IG particles in five partitions of perimeters of sections constituting segments of swellings with (**F**; *N* = 14) or without (**G**; *N* = 37) an inhibitory bouton in their proximity. Each data point represents the sum of IG particles found in one of the partitions of sections constituting a segment (see schemes in insets). Mean values are indicated by horizontal bars. The quantity of IG particles in each partition was normalized to the total number of the particles in a segment. The partitions are labeled 1–5 while that containing a reference point (blue in each inset) is the partition #1. The reference point was set either as an intersection of the calyceal membrane and a link between midpoints of cross-sections through a swelling and a bouton **(F)** or at the horizontal edge of a swelling **(G)**. Note significantly increased amounts of IG particles in the partition adjacent to the inhibitory bouton. *** *P* < 0.001; ** *P* < 0.01; * *P* < 0.05; Dunnett’s multiple comparison test (data from the partition #1 were used as a control).

High-frequency stimulation of glycinergic fibers evokes strychnine-sensitive facilitation of glutamate release from the calyx (Turecek and Trussell, 2001). The heterosynaptic nature of the facilitation led to the proposition that it results from presynaptic GlyR activation by glycine spillover. This mechanism of activation requires the receptors to be located at sites accessible by ambient agonist (Rusakov et al., 1999). Consistent with this assumption, findings in our study (Figure 5) indicated GlyRs on the side of the calyx that is exposed to the extracellular space. To provide further insight into the mechanism of presynaptic GlyR activation by endogenous glycine, we analyzed the distribution of the receptors in swellings adjacent to glycinergic boutons. Figures 6A,B show numerous IG particles in calyceal parts proximal to putative inhibitory terminal (vGluT1-negative structure that contains pleomorphic synaptic vesicles and forms synaptic contacts with a PC). Using 3D reconstructed segments we measured the spatial distances between each IG particle and the middle of the PSD under the inhibitory AZ (see scheme in Figure 6D). The cumulative probability histogram of these distances indicates that most of IG particles lie within ~1.5 µm from the AZ, the distance that roughly matches a small molecule transmitter spillover range (Faber and Korn, 1988; Barbour and Hausser, 1997; Figure 6C). Interestingly, a comparison between the distribution of IG particles and the distribution of contoured surface points of the segments showed a significant difference (Kolmogorov-Smirnov test, *P* < 0.001) (Figure 6D) and suggested a more frequent incidence of GlyRs in parts closer to the inhibitory AZ. Similar differences were not observed in swellings without glycinergic terminals in their vicinity (Figure 6E) implying that the surface distribution of GlyRs is affected by the presence of an endogenous agonist.

This hypothesis was further tested by comparing the IG particle distributions along perimeters of calyceal cross-sections in the presence or absence of the inhibitory terminals. Perimeters of sections constituting swellings with or without the inhibitory terminals were similar (7.2 ± 3.1 µm vs. 7.5 ± 3.9 µm; 25%/75% percentiles: 4.6/9.8 µm vs. 4.8/9.3 µm). We therefore analyzed the distribution of IG particles among membrane regions that were proportional to the perimeter length. The perimeter of every section was subdivided to five even intervals and the number of IG particles was counted in each of them (see schemes in Figures 6F,G). The data showed a significantly increased amount of particles in a partition adjacent to the inhibitory terminal (Figure 6F). In sections without an identified inhibitory bouton this localization pattern was not observed (Figure 6G). Thus the data shows that the surface distribution of presynaptic GlyRs is consistent with the activation of the receptors by glycine spillover from inhibitory terminals. Moreover, the data suggests that the distribution of the receptors in calyceal processes depends on the presence of glycinergic nerve terminals. The average density of IG particles in swellings with or without an inhibitory bouton in their proximity was not significantly different (11.9 ± 8.3 IG/µm^2^ vs. 10.0 ± 7.4 IG/µm^2^; unpaired Student’s *t*-test). This indicated that the presence of inhibitory AZ affects the location of GlyRs rather than their overall expression level in calyceal swellings.

## Discussion

In this study we analyzed the distribution of GlyR-associated IG particles in the mature calyx of Held nerve terminal. To discriminate between the presynaptic and postsynaptic labeling, we used antibodies that recognize an intracellular part of the receptor (Hruskova et al., 2012). Our data suggests that the locations of presynaptic GlyRs correlate with both the function of the receptors and their accessibility for endogenous agonists. We found the strongest labeling in calyceal swellings, compartments releasing glutamate and characterized by high density of voltage-gated Ca^2+^ channels (VGCC; Spirou et al., 2008). This observation would be thus consistent with the role of the receptors in Ca^2+^-dependent enhancement of the presynaptic release probability by glycine (Turecek and Trussell, 2001; Awatramani et al., 2005). Calyceal GlyRs, however, do not preferentially locate near the glutamate release sites in domains typical for analogous presynaptic autoreceptors. These include ionotropic glutamate receptors that form Ca^2+^-permeable channels able to facilitate exocytosis of glutamate by delivering Ca^2+^ directly into the presynaptic AZ (see Engelman and MacDermott, 2004; Pinheiro and Mulle, 2008; Ruiz and Kullmann, 2013 for review). GlyRs elevate presynaptic Ca^2+^ concentration indirectly, via chloride efflux leading to nerve terminal depolarization and subsequent activation of presynaptic VGCC (Turecek and Trussell, 2001; Price and Trussell, 2006; Huang and Trussell, 2008). Moreover, the mechanism downstream of the presynaptic calcium signals was observed to include protein kinase C-dependent enhancement of the number of the readily releasable vesicles (Chu et al., 2012). Hence, the synaptic location of GlyRs does not seem to be strictly required for an effective modulation of glutamate release. In fact, the extrasynaptic incidence of GlyRs acting via a cascade of signaling events might be expected as the time course of glycine-induced facilitation is much slower than that of presynaptic GlyR currents (Turecek and Trussell, 2001). In contrast, the facilitatory effect of GlyRs on glycinergic boutons at dissociated spinal cord neurons appears to have a faster time course (Jeong et al., 2003), suggesting a more direct mechanism operating closer to the glycine release sites. Thus the subcellular distributions of GlyRs on glutamatergic and glycinergic terminals likely differ and match the common distribution patterns proposed for presynaptic heteroreceptors vs. autoreceptors (Pinheiro and Mulle, 2008).

Heterosynaptic modes of activation have been well documented for presynaptic GABA-A receptors inducing facilitation of glutamate release from hippocampal mossy fibers (Ruiz et al., 2003; Alle and Geiger, 2007). GlyRs, analogously operating at the calyx of Held synapse, could therefore also be activated by agonists diffusing from the inhibitory release sites. In agreement with this hypothesis, a repetitive stimulation of glycinergic fibers innervating MNTB PC was observed to enhance the release of glutamate from the calyx (Turecek and Trussell, 2001). Moreover, here we show that GlyRs preferentially occupy those parts of calyceal swellings that are not in contact with the postsynaptic cell body and that the receptors tend to align close to glycinergic boutons. Thus the spatial distribution of the receptors seems to be well adjusted to sense the extracellular agonist concentration dropping as a cubic function of the distance from the inhibitory AZ (Vizi et al., 2010). At this point it is not clear whether such activation of calyceal GlyRs is under the control of glycine transporters, similarly to how presynaptic GABA-A spillover currents recorded from the mossy fibers are shaped by the GABA uptake system (Alle and Geiger, 2007). The sequestration of glycine might indeed be expected as MNTB cells are strongly immunopositive for type 1 and 2 glycine transporters (GlyT1 and GlyT2; Zafra et al., 1995; Friauf et al., 1999; Zeilhofer et al., 2005). Glia-specific GlyT1 might be of particular interest as calyces are surrounded by astrocytic processes and GlyT1 inhibitors have been found to affect a sound-evoked activity in gerbil MNTB (Sätzler et al., 2002; Kopp-Scheinpflug et al., 2008; Reyes-Haro et al., 2010; Uwechue et al., 2012). The reversal of the glial uptake of glycine might in turn activate those calyceal GlyRs that could not be reached by the spillover from glycinergic terminals.

The non-homogenous distribution of presynaptic GlyRs spatially related to inhibitory release sites suggests the presence of the use-dependent mechanism of receptor relocation. It is well established that excitatory synaptic activity can exert changes in the subcellular distribution of ionotropic glutamate receptors via numerous molecular mechanisms (reviewed by Triller and Choquet, 2005; Newpher and Ehlers, 2008; Gladding and Raymond, 2011). Likewise, an elevation in cytoplasmic Ca^2+^ concentration has been shown to stimulate GlyR accumulation at postsynaptic sites via a mechanism involving protein-protein interactions with synaptic scaffolds (Lévi et al., 2008). The interactions largely build upon the GlyR β subunit-binding protein gephyrin which reduces the lateral diffusibility of heteromeric GlyRs and connects them to cytoskeletal components (Kneussel and Betz, 2000; Meier et al., 2001). Gephyrin was not found in the calyx of Held terminal (Hruskova et al., 2012), indicating relatively mobile presynaptic α1 homomeric GlyRs (Turecek and Trussell, 2002; Hruskova et al., 2012; Xiong et al., 2014) with a potential of use-dependent redistribution by gephyrin-independent mechanisms. More experiments would be needed to find out whether these processes involve receptor-stimulated sorting of presynaptic membrane to specialized zones with subsets of GlyRs confined in their lateral movement (Sheets et al., 1995; Kusumi et al., 2012). Such activity-regulated changes in presynaptic GlyR distribution could underlie plasticity of glycine-mediated signaling in MNTB.

## Conflict of interest statement

The authors declare that the research was conducted in the absence of any commercial or financial relationships that could be construed as a potential conflict of interest.

## Footnotes

^1^http://rsbweb.nih.gov/ij/
